# A golden anniversary: highlights of the 50th annual meeting of the American Society of Clinical Oncology

**DOI:** 10.3332/ecancer.2014.444

**Published:** 2014-07-10

**Authors:** Gordon McVie, Audrey Nailor

**Affiliations:** 1 European Institute of Oncology, Via Ripamonti, Milan 20141, Italy; 2 Cancer Intelligence Ltd, Bristol BS6 5RL, UK

**Keywords:** personalised medicine, immunotherapy, hormone-sensitive cancers

## Abstract

The 50th Annual Meeting of the American Society of Clinical Oncology showed a shift in the culture of cancer research, moving towards multidisciplinary, integrated, and patient-centric work. Hormone-sensitive cancers were particularly highlighted at this meeting, and impressive strides were made in the previously underserved areas of the lung and thyroid cancer. Interestingly, immunotherapy was one of the strongest themes to emerge.

## Introduction

Over 32,000 participants attended the 50th Annual Meeting of the American Society of Clinical Oncology (ASCO), held in Chicago, Illinois, United States. One of the largest and most respected cancer conferences, the meeting attracts a broad range of scientists, health-care professionals, and patient advocates from all over the world. This conference report will summarise the key abstracts of the meeting.

The theme of the meeting was ‘Science and Society: The Next 50 Years’. ASCO has just produced the inaugural report on The State of Cancer Care in America, highlighting the rising costs of cancer care as well as its poor accessibility. The changing health-care system of the United States, and the resulting preoccupation with funding at ASCO, was offset by greater global strides in cancer research and development.

This year, hormone-sensitive cancers were a particularly energising topic, with new research in breast, prostate, and gynaecological cancers indicating a bright future for hormonal agents. Targeted treatments, such as hormonal agents and anti-angiogenesis drugs, enjoyed a rising swell of excellent research, while combination therapies remain some of the most popular topics of clinical study. However, what most attendees commented on was the strong theme of immunotherapy, which dominated the conversation.

All in all, this was a high-quality meeting of ASCO, with familiar faces and rising stars presenting some interesting new data.

## Hormone-sensitive cancers: the talk of the town

The hormone-sensitive cancers were of the greatest interest in this year’s ASCO, with several studies in prostate cancer attracting particular attention.

An abstract presented by Professor Christopher Sweeney of the Dana-Farber Institute [2] showed that in adjuvant treatment of prostate cancer, docetaxel, in combination with androgen-deprivation therapy, demonstrated significantly better results than docetaxel alone. While these results are preliminary, they suggest that neurologists and medical oncologists can look forward to multidisciplinary approaches to prostate cancer.

The two ‘new kids on the block’ in castration-resistant prostate cancer received some attention. Abiraterone acetate and enzalutamide both appeared to stand up to the publicity generated last year, and were feted again at this year’s ASCO. It appears that neither drug is preferable in second-line therapy, and both are suited for the first-line approach.

A combined study of abiraterone plus enzalutamide, led by Professor Eleni Efstathiou of the MD Anderson Cancer Centre [3], promised a new way forward for patients with metastatic castration-resistant prostate cancer. This combination appears both safe and effective, with no drug-related deaths and significant improvement in patient outcomes. While the study was limited by its small size, the results are very promising. Altogether, these studies show great strides being made in castration-resistant prostate cancer—a previously underserved field.

In contrast, two large, well-anticipated breast cancer studies from the International Breast Cancer Study Group were somewhat disappointing. One study found that lapatinib produces insignificant extra effects in combination with trastuzumab. The other large study showed that exemestane, an aromatase inhibitor, was superior to Tamoxifen in younger women with hormone-sensitive breast cancer combined with ovarian suppression. However, Exemestane remains costly. While these results are important in Western countries where Exemestane may be affordable, it appears that Tamoxifen will remain the gold standard globally.

On the topic of ovarian suppression, LHRH analogue suppression of the ovaries with goserelin showed encouraging effects in protecting the ovaries in premenopausal women receiving chemotherapy [4].

## Lung cancer: small-cell gains ground

Small-cell lung cancer made a significant showing at ASCO for the first time in 20 years. A study demonstrated that even in extensive disease, irradiating the primary tumour had an effect even on distant metastases.

Dr Yuichi Ozawa led an interesting study demonstrating that primary cranial irradiation (PCI) was unnecessary in small-cell lung cancer without visible metastases on an MRI scan [5]. This is very positive news for patients who have achieved MRI-confirmed remission of small-cell lung cancer, potentially sparing them the toxicity of prophylactic cranial irradiation.

A number of new drugs are being tested in advanced non-small-cell lung cancer; however, none are currently poised to impress. One notable finding, brought to us by Dr Maurice Pérol, is that the new anti-angiogenesis drug, ramucirumab, in combination with docetaxel, extends survival in advanced non-small-cell lung cancer. This extent of survival is unusual in this disease, and very heartening.

## Ovarian cancer: anti-angiogenesis the new platinum standard?

In the past few years, anti-angiogenesis drugs have performed well in ovarian cancer, providing some good news for patients who are platinum-resistant.

ASCO 2014 saw the anti-angiogenesis drug cedirumab being tested in combination with PARP inhibitor olaparib. Led by Dr Joyce Liu of the Dana-Farber Cancer Institute, the study found that the combination was more positive for patients’ progression-free survival than olaparib alone.

## Colorectal cancer: a pause in movement

While colorectal cancer is now the third most common cancer, there were relatively few abstracts reflecting this at ASCO.

One important clarification will serve oncologists who frequently see patients with colorectal cancer. At this year’s ASCO, oncologists agreed that there is relatively little difference between the targeted chemotherapy regimens FOLFIRI and FOLFOX. Professor Alan Venook of the University of California presented a large federally funded phase III study comparing bevacizumab with chemotherapy against cetuximab with chemotherapy, showing that there is little difference between them. Thus, the suitable combinations should be chosen according to the patient’s status and other comorbidities, offering some flexibility for personalised medicine.

## Melanoma: good news ahead

Melanoma made headlines in the 2012 ASCO, with ipilimumab, an innovative immunotherapy, generating much attention. At this year’s ASCO, PD-1 inhibitors received similar accolades, suggesting an exciting future ahead for immunotherapeutic melanoma treatments.

Having shown such spectacular success in metastatic melanoma, ipilimumab has been moved up front as an adjuvant in resectable melanoma; there was significant progression-free survival data to consider at the meeting. Professor Alexander Eggermont, of the Gustave Roussy Cancer Campus Grand Paris, presented the first study demonstrating ipilimumab’s effectiveness as an adjuvant therapy in earlier disease onsets. In high-risk stage III melanoma, adjuvant ipilimumab decreased the risk of cancer recurrence by 25% against the placebo.

The new PD-1 inhibitors are arriving as potential challengers to ipilimumab. The PD-1 targeting antibody MK-3475 was applauded at this ASCO, particularly after a key study led by Professor Antoni Ribas of the David Geffen School of Medicine at the University of California. In cases where advanced metastatic melanoma progressed after treatment with ipilimumab, treatment with MK-3475 showed a high percentage of long-term survival with durable response rates. MK-3475, previously named lambrolizumab, is now to be named pembrolizumab.

But more effective than a simple challenge is combination therapy. It seems that ipilimumab and PD-1 checkpoint inhibitor drugs are indeed hitting different targets, and may be complementary in combination. This was demonstrated nicely in a study presented by Professor Mario Sznol, combining nivolumab with ipilumumab in patients with advanced melanoma. With a two-year survival rate of 79%, the results are very encouraging.

## Rare blood cancers: big stories emerging

One paper on chronic lymphocytic leukaemia (CLL) particularly stood out in the plenary sessions. Professor John C. Byrd, of The Ohio State University Comprehensive Cancer Center, showed that ibrutinib, a Bruton’s tyrosine kinase inhibitor, is an improvement over standard treatment for aggressive relapsed resistant CLL. He stated that over 80% of patients treated with ibrutinib were in remission after one year, compared to about 40% in remission with ofatumumab—essentially doubling the effectiveness of treatment.

While mantle cell lymphoma remains a rare blood cancer, a new and effective drug cocktail has greater implications. Bortezomib has proven to be a safe and effective drug with few side effects, suitable for patients with a median age of around 70. A randomised trial presented by Dr Franco Cavalli of the Oncology Institute of Southern Sweden compared the standard treatment, rituximab-CHOP, with a cocktail in which the standard vincristine component was replaced with bortezomib. The replacement demonstrated a 59% improvement in progression-free survival when compared to the standard. This modified cocktail is expected to show effectiveness in the more common B-lymphocyte lymphoid neoplasms.

## Thyroid cancer: black sheep gets screen time

Thyroid cancer is frequently treated by radiation medicine divisions or nuclear medicine divisions in hospitals, as most thyroid cancers, even when metastatic, respond well to injections of radioactive iodine. For patients who are in the minority and who are resistant to radio-iodine or cytotoxics, there is lenvatinib, a new drug reported by Dr Martin Schlumberger of the Gustave Roussy team in France.

In a randomised trial of lenvatinib versus standard care for refractory differentiated thyroid cancer, there was dramatic improvement in all the outcomes measured, with response rates over the 50% mark (compared to 5-10% response in standard treatment) and progression-free survival of two to three years versus less than six months. Although thyroid cancer is a rare and underserved cancer, it’s very important that this targeted drug can produce such impressive anti-cancer responses.

## Conclusions

The golden anniversary of ASCO reminds us that these are exciting times to be involved in cancer research. Fifty years ago, the concept of using the body’s own immune system to combat cancer was far from reality. Now, immunotherapy is established as a viable and effective means of treating cancer. Equally, treatments that extend survival in aggressive cancers demonstrate increasing hope for cancer patients. Patient-centred care, from personalised medicine for fertility-sparing treatments, informs research approaches more than ever.
